# Thrombin Generation in Zebrafish Blood

**DOI:** 10.1371/journal.pone.0149135

**Published:** 2016-02-12

**Authors:** Evelien Schurgers, Martijn Moorlag, Coenraad Hemker, Theo Lindhout, Hilde Kelchtermans, Bas de Laat

**Affiliations:** 1 Cardiovascular Research Institute Maastricht, Maastricht University Medical Centre, Maastricht, The Netherlands; 2 Synapse BV, Maastricht, The Netherlands; IIBB-CSIC-IDIBAPS, SPAIN

## Abstract

To better understand hypercoagulability as an underlying cause for thrombosis, the leading cause of death in the Western world, new assays to study *ex vivo* coagulation are essential. The zebrafish is generally accepted as a good model for human hemostasis and thrombosis, as the hemostatic system proved to be similar to that in man. Their small size however, has been a hurdle for more widespread use in hemostasis related research. In this study we developed a method that enables the measurement of thrombin generation in a single drop of non-anticoagulated zebrafish blood. Pre-treatment of the fish with inhibitors of FXa and thrombin, resulted in a dose dependent diminishing of thrombin generation, demonstrating the validity of the assay. In order to establish the relationship between whole blood thrombin generation and fibrin formation, we visualized the resulting fibrin network by scanning electron microscopy. Taken together, in this study we developed a fast and reliable method to measure thrombin generation in whole blood collected from a single zebrafish. Given the similarities between coagulation pathways of zebrafish and mammals, zebrafish may be an ideal animal model to determine the effect of novel therapeutics on thrombin generation. Additionally, because of the ease with which gene functions can be silenced, zebrafish may serve as a model organism for mechanistical research in thrombosis and hemostasis.

## Introduction

Thrombosis remains a leading cause of death in the western world. Aside from mortality, significant morbidity occurs from thrombotic events. The causes of this hypercoagulability are becoming more and more clear with an enhanced knowledge of hemostasis and the development of new coagulation assays. Most of this knowledge results from extensive *in vitro* biochemical characterization of blood coagulation, whereas studies investigating blood coagulation *in vivo* are limited.

Due to the availability of knockout technology, genetic studies of thrombosis in mice are popular. Nonetheless, the time consuming and labor-intensive process of generating knockouts restricts these studies. The zebrafish is generally accepted as a good model for mammalian hemostasis and thrombosis due to the presence of coagulation factors, platelet receptors and its response to anti-coagulant drugs commonly used in clinical treatment [1]. In addition, hemostatic pathways in zebrafish proved to be similar to those in man [2–4]. Interestingly, the use of zebrafish enables large scale mutagenesis screening to identify novel genes involved in hemostasis and thrombosis [5–7].

The small size of zebrafish has been a hurdle in thrombosis and hemostasis research since most of the conventional coagulation assays require large amounts of plasma. Jagadeeswaran *et al*. optimized a total coagulation activity screening assay using small quantities of zebrafish plasma, by adding human fibrinogen and measuring fibrin formation by turbidimetry to probe thrombin formation [8]. Additionally, they developed an ultra-sensitive kinetic method to identify specific pathway defects in small quantities of plasma. Disadvantages of such assays include the requirement of multiple zebrafish and the use of plasma, not taking into account the effect of thrombocytes and erythrocytes on coagulation.

At present, mainly end-point assays are used to detect coagulation defects. These assays simply measure the time it takes for a platelet-poor plasma sample to clot, i.e. when the first traces of fibrin are formed. However, fibrin formation already starts in the presence of tiny amounts of thrombin (≈1 nM) [9]. Thus, the vast majority of thrombin generation takes place after fibrin formation [10], suggesting that clotting time-based assays only measure the initiation and not the propagation phase of coagulation. Importantly, correct functioning of the hemostatic system proved to be dependent on the total amount of thrombin that is formed during coagulation [11]. We recently developed a reliable method to measure thrombin generation in a drop of whole blood, thereby bringing coagulation one step closer to physiology [12]. In this study, we further optimized this method enabling the determination of thrombin generation in a drop of non-anticoagulated whole blood obtained from a single zebrafish. Thrombin generation proved to be sensitive to pre-treatment of the fish with inhibitors of FXa and thrombin. Furthermore, we visualized the resulting fibrin network by scanning electron microscopy (SEM) in order to analyze the density and dimensions of the fibrin strands.

## Materials and Methods

### Reagents

20 mM Hepes buffer (pH 7.35) containing 5 mg/ml bovine serum albumin (BSA) and 140 mM NaCl (BSA5) or 60 mg/ml bovine serum albumin (BSA60) were prepared as described previously [13]. The rhodamine-based substrate P_2_Rho was a kind gift of Diagnostica Stago. The calibrator, α_2_macroglobulin-thrombin (α_2_M-T) complex was prepared in-house as described previously [13]. Rivaroxaban (Xarelto) was from Bayer and melagatran was a gift from AstraZeneca.

### Blood collection and treatment of zebrafish

This study was carried out in strict accordance with the recommendations in the guide for the use of laboratory animals of the university of Liège. The protocol was approved by the committee on the ethics of animal experiments of the university of Liège Permit number LA 1610002. Blood was collected from adult (male and female) wild type zebrafish (*Danio rerio)* as described previously [8]. Briefly, fish were sedated by immersion in ice water. Subsequently, with a small pair of scissors, an incision was made at the lateral side of the fish just posterior of the dorsal fin, thereby transecting the dorsal vein/artery. From the blood welling up in the wound, 5 μl was collected for further analysis.

For anticoagulant treatment, fish were sedated with tricaine (0.16 mg/ml). Fish were dried with paper and weighed. Only fish weighing less than 1 gram were used and injected intraperitoneally with 20 μl/g of the indicated anticoagulant in phosphate buffered saline (PBS) and 0.25% phenol red (to monitor the injection process). After injection, fish were allowed to recover for 30 minutes after which blood was collected as described above.

### Thrombin generation measurement

For thrombin generation, an adapted protocol was developed based on our whole blood thrombin generation assay [12]. Collected whole blood (5 μl) was mixed with 5 μl of HEPES buffer containing the P_2_Rho substrate (final concentration (fc) 300 μM). 5 μl of this mixture was put on a paper disk and covered with mineral oil to prevent evaporation. The lag time phase of the thrombin generation experiment was started as soon as the incision for the blood withdrawal was made. Calibration was done by adding 5 μl of whole blood to 5 μl of HEPES buffer containing P_2_Rho (fc 300 μM), α2M-thrombin calibrator (fc 100 nM) and citrate (fc 9,8 mM). Fluorescence was recorded with a fluorescence detector (ESElog, Qiagen) with γ_ex_ = 485 nm and γ_em_ = 538 nm. All experiments were performed at 37°C, unless stated otherwise.

Analysis of the fluorescence tracings to yield the thrombogram and corresponding parameters was performed with a modified method, taken into account only the thrombin generation until the peak is reached. From the resulting thrombogram the following parameters were calculated: lag time (min), peak (nM, maximal thrombin concentration), peak-endogenous thrombin potential (ETP, nM.min, area under the thrombin curve until the peak is reached), time to peak (min) and velocity (nM/min, maximal rate of thrombin generation).

The human plasma samples were analyzed using the plasma calibrated automated thrombography (CAT) as previously described [13] in the presence or absence of either rivaroxaban or melagatran.

### Plasma samples for normal pool plasma

After approval of the local medical ethical board (Medical Ethical Committee of Maastricht University Medical Center) 24 healthy adult volunteers who did not take any drugs for at least two weeks gave full informed written consent according to the Helsinki declaration. Blood was collected aseptically by antecubital puncture into vacuum tubes (1 volume trisodium citrate 0.105M to 9 volumes blood) (BD Vacutainer System).

For the normal pooled plasma (NPP), blood from the 24 volunteers was prepared by centrifuging the blood at 2900g during 10 min at room temperature. Plasma was aspirated and the procedure was repeated. Plasmas were pooled and further ultra-centrifugation (100000g, 70 min) was carried out. Aliquots of 1 ml were stored at -80°C until use.

### SEM analysis

After thrombin generation was determined in whole blood, the clots were prepared for visualization by SEM. The clots were fixated by adding 2.5% glutaraldehyde (grade I, Sigma Aldrich, St. Louis, Missouri) in PBS (Sorensen’s, pH 7.2) (Electron Microscopy Sciences, Hatfield, PA, USA) for 1 hour at room temperature and then placing it at 4°C overnight. The following day, the glutaraldehyde solution was removed and the samples were repeatedly (5x) washed with PBS. As a secondary fixation, the samples were placed in osmiumtetroxide (OsO_4_, 1%) diluted in sodium cacodylate (200 mM, pH 7.4) (Electron Microscopy Sciences, Hatfield, PA, USA) for 1 hour at room temperature. Consecutively, the clots were dehydrated in ethanol (30%, 50%, 70%, 90% and 3 times at 100%) for 3 minutes. The samples were then treated with a hexamethyldisilazane/ethanol solution for 3 minutes and in hexamethyldisilazane (Sigma Aldrich, St. Louis, MO, USA) for 10 minutes. The samples were removed from the wells, left to dry and coated with gold. Analysis was performed on a desktop SEM (Phenom-World, Eindhoven, the Netherlands).

### Statistical analysis

For statistical analysis of the data GraphPad Prism Software was used. Differences between two groups were evaluated by the one-way ANOVA test or the Mann Whitney U test.

## Results

### Development of an assay to measure thrombin generation in zebrafish

In a first set of experiments, thrombin generation was determined in 5 μl whole blood collected from single zebrafish, using a method comparable to whole blood calibrated automated thrombography [12]. The limited blood volume of a single zebrafish impedes the performance of both a thrombin generation and calibrator measurement. Since calibrator measurements performed on the blood of 28 different fish in 5 independent experiments demonstrated an acceptable variation (mean CV of 14%), the average calibrator slope per experiment was used for thrombin generation calculations.

**Fig 1:**
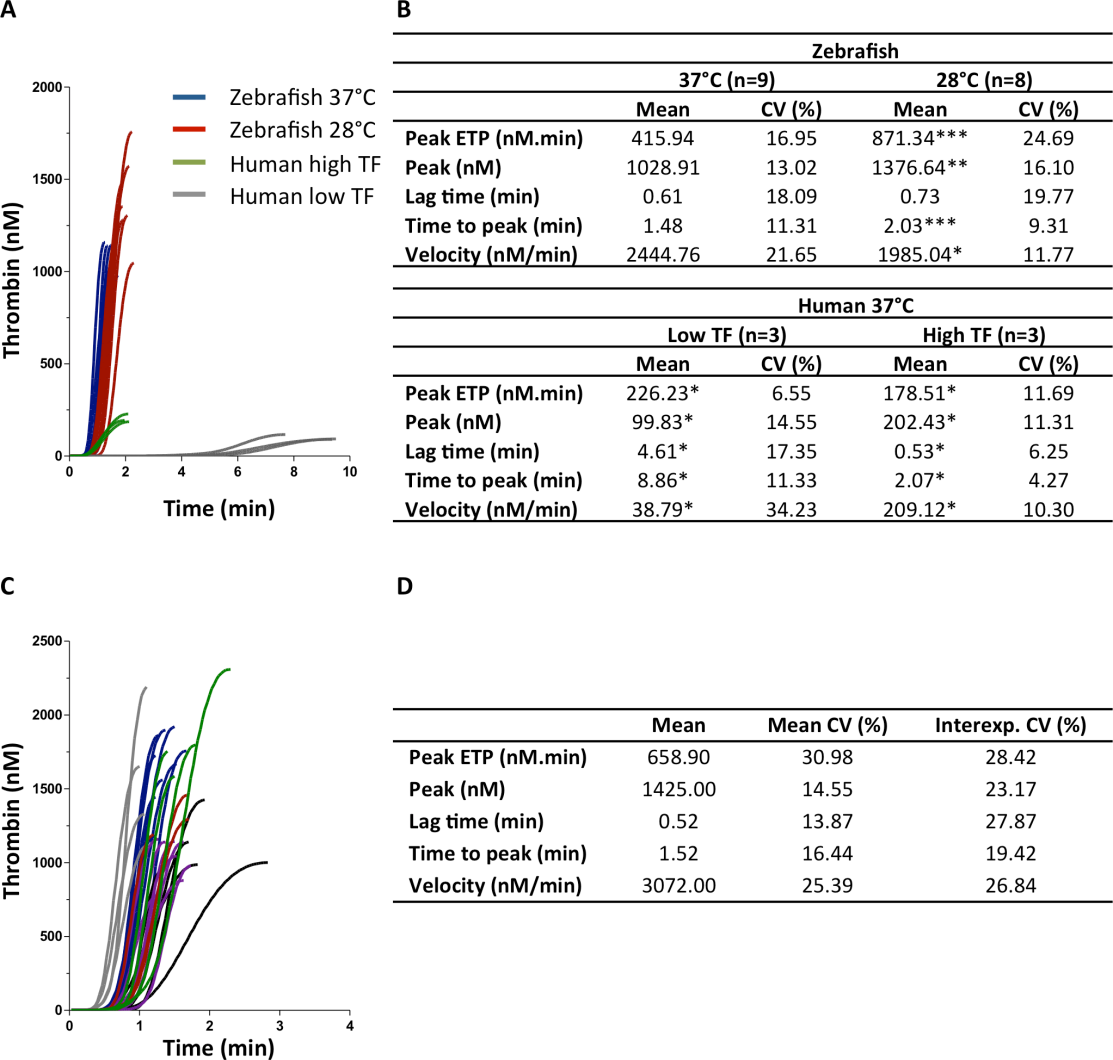
Thrombin generation curves and parameters in zebrafish, effect of temperature and comparison with humans. Thrombin generation was measured in zebrafish at 28°C and 37°C and compared to thrombin generation in humans at 37°C with low (no tissue factor (TF) added) or high (100 pM) TF. **(A)** Thrombin generation curves are shown (n = 8 or 9 for fish, n = 3 for humans). **(B)** Mean thrombin generation parameters and their CV are calculated. Statistical differences as compared to the zebrafish group measured at 37°C are indicated (*p<0.05, **p<0.01, ***p<0.0001, 1-way ANOVA). **(C-D)** Thrombin generation was measured in 6 independent experiments, in a total of 34 zebrafish. **(C)** Thrombin generation curves are shown. Each color represents an independent experiment. **(D)** Mean thrombin generation parameters and their CV are calculated, as well as the CV between the means of all 6 experiments (interexp. CV).

The thrombin enzyme from zebrafish clearly proved to be capable of cleaving our rhodamine-based substrate. Thrombin generation experiments on zebrafish blood performed at 37°C or 28°C (physiological temperature of zebrafish) demonstrated significant differences between the two temperatures. (Fig 1A and 1B). As previously described for human plasma [14], a lower temperature resulted in slower and higher thrombin generation. Due to practical considerations and because the adapted PT and APTT for zebrafish plasma by Jagadeeswaran *et al*. are performed at 37°C [4], we decided to perform all subsequent experiments at 37°C. For comparison with human thrombin generation, blood was collected from a healthy volunteer by finger prick. When thrombin generation was measured without added tissue factor (TF), a much slower and lower thrombin generation was observed as compared to zebrafish. When a high amount of TF (100 pM) was added to the human blood, thrombin generation was accelerated, but the striking differences with zebrafish thrombin generation were still apparent as demonstrated by significant differences in ETP, peak, lag time and velocity (Fig 1A and 1B).

Subsequently, thrombin generation was measured in 6 independent experiments, each consisting of 3 to 9 thrombin generation measurements. Thrombin generation curves, together with the average and %CV of the individual thrombin generation parameters are shown in Fig 1C and 1D. Interestingly, thrombin generation measured in individual fish showed a similar amount of inter-individual variation as in humans [12]. Also the interexperimental variation between these 6 experiments was calculated and proved to be acceptable (Fig 1D). Taken together, our results demonstrate the feasibility of measuring thrombin generation in whole blood collected from a single zebrafish.

**Fig 2:**
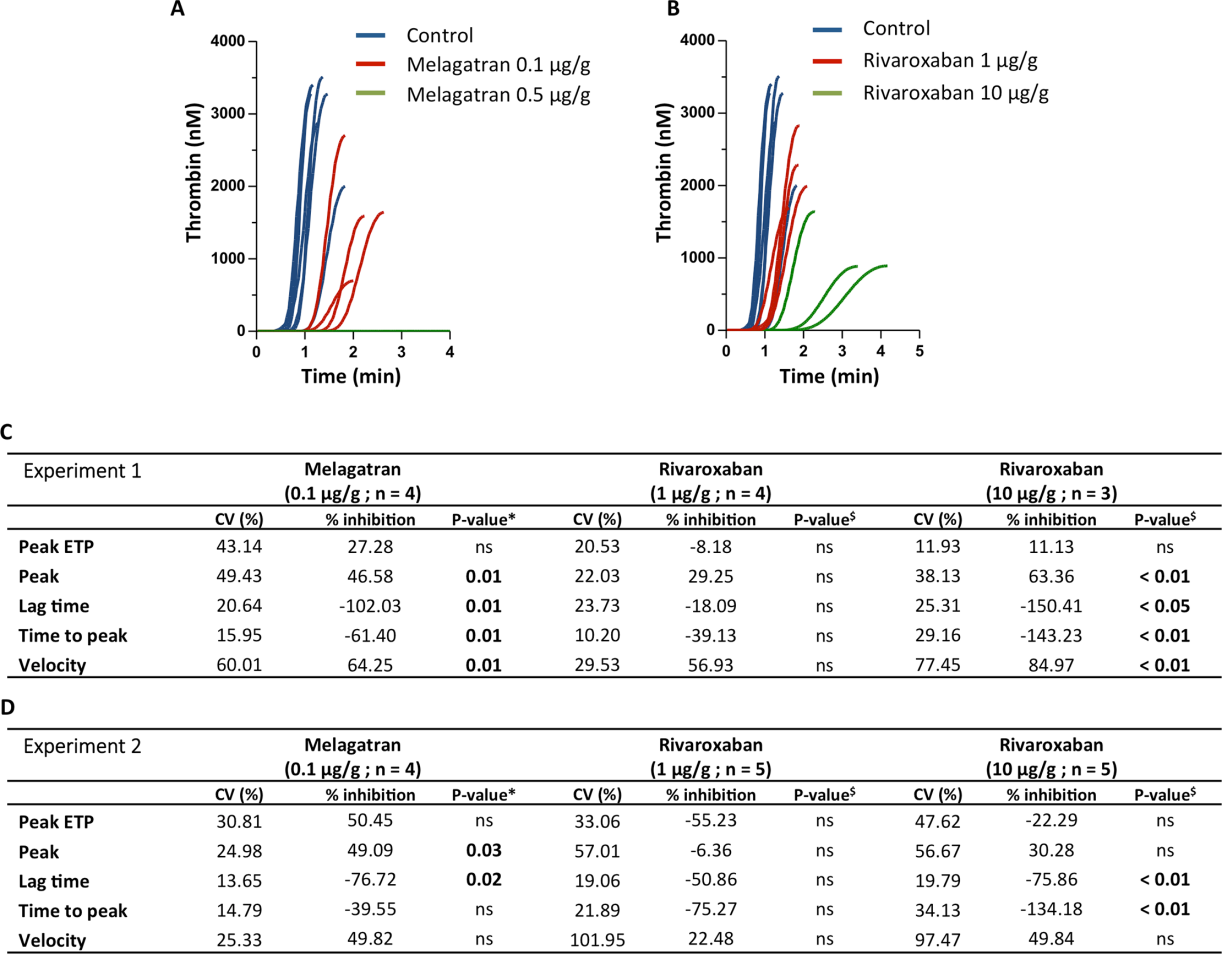
Inhibition of thrombin generation in zebrafish by thrombin or FXa inhibition. Zebrafish were treated with melagatran **(A)** or rivaroxaban **(B)** at the indicated doses for 30 min, followed by thrombin generation measurements. **(C-D)** Results of two independent experiments are expressed as the percentage inhibition compared to a control group of vehicle-treated fish (n = 8). The percentage CV is indicated for all parameters. Statistical differences between treatment and control groups were performed with *Mann-Whitney U-test or ^$^1-way ANOVA. (ns = not significant).

In a next set of experiments, the effect of pre-treatment of the zebrafish with thrombin or FXa inhibitors on thrombin generation parameters was tested. In two independent experiments, zebrafish were treated with the indicated compounds and doses for 30 minutes as mentioned before, followed by blood collection and thrombin generation measurements. Thrombin generation curves of the individual zebrafish of one experiment are shown in Fig 2A and 2B. Results of the two independent experiments are expressed as the percentage inhibition compared to a control group of vehicle-treated fish (n = 8) in Fig 2C and 2D.

As to the thrombin inhibition, treatment with melagatran concentrations above 0.5 μg/g completely blocked thrombin generation, clearly illustrating that the observed fluorescent signal is thrombin- related (Fig 2A). A concentration of 0.1 μg/g melagatran significantly inhibited thrombin generation, as evident from the significantly decreased peak height and velocity index and increased lag time and time to peak (Fig 2A–2C and 2D). Comparable results were obtained upon injection of another thrombin inhibitor, hirudin (4 μg/g). Hirudin treatment resulted in a significant (p < 0.03) inhibition of the peak ETP, peak and velocity by 78%, 85% and 90% respectively. Lag time and time to peak were increased by 329% and 238% respectively (p = 0.06). Inhibition of FXa by rivaroxaban treatment demonstrated a dose-dependent inhibition of thrombin generation, with a dose of 10 μg/g significantly inhibiting thrombin generation (Fig 2B, 2C and 2D).

For melagatran, the dosage needed to inhibit thrombin generation in zebrafish was comparable to concentrations used in *in vitro* experiments with human normal pooled plasma, where 0.22 μg/ml gave a 20% and -498% inhibition on the peak height and lag time respectively. A dose of 0.43 μg/ml resulted in 95% inhibition of the peak and -1297% of the lag time. For rivaroxaban, the dosage needed for inhibition in fish was higher than dosages needed to inhibit thrombin generation *in vitro* in human normal pooled plasma. A dosage of 0.44 μg/ml gave a 95% and -1297% inhibition on the peak height and lag time respectively. However, the dosages used in the zebrafish, both of melagatran and rivaroxaban, were much closer to the prescribed dose for adults of 20 mg/day (± 0.29 μg/g).

**Fig 3:**
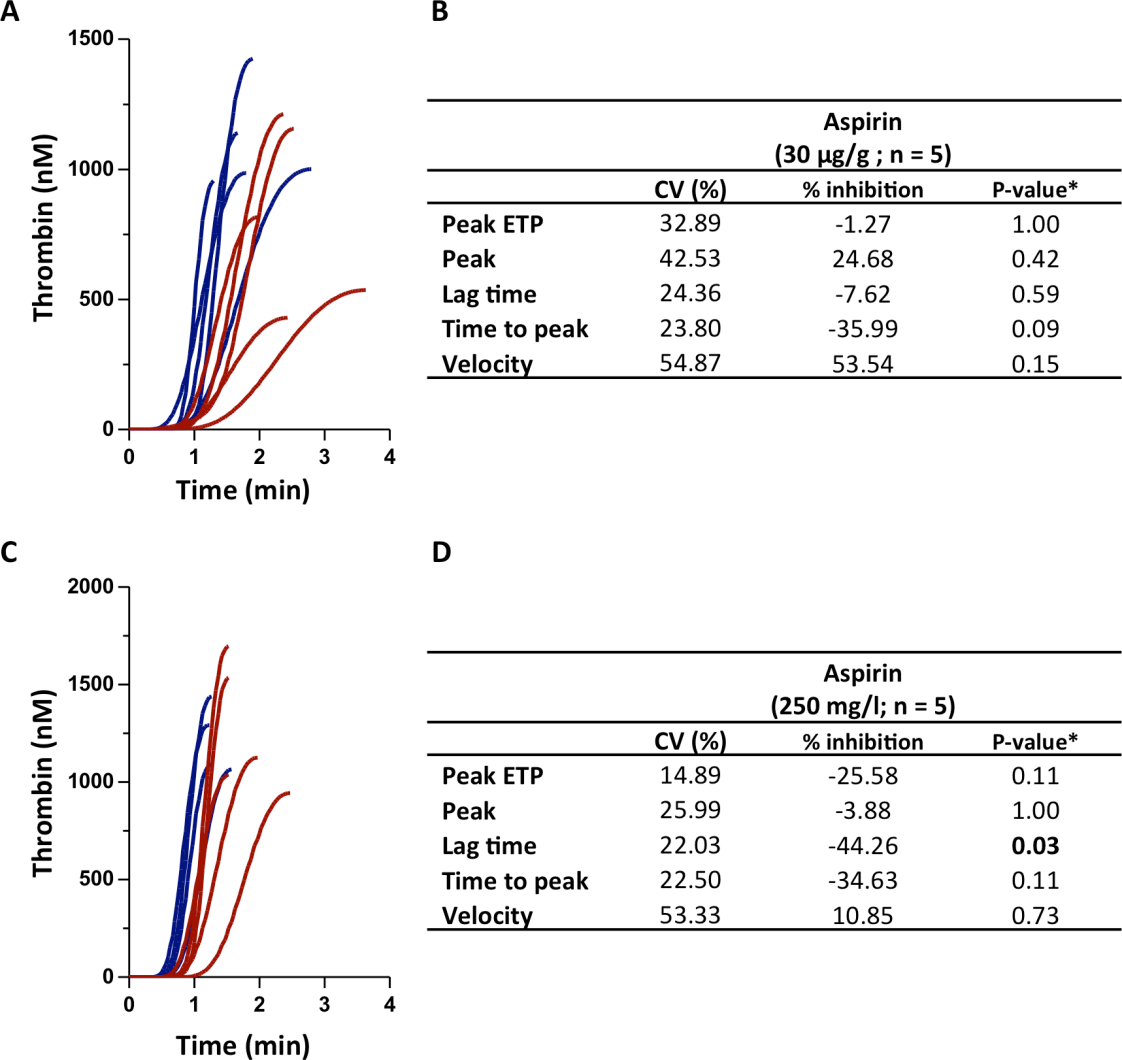
Inhibition of thrombin generation in zebrafish by inhibition of platelet aggregation. Zebrafish were treated with 30 μg/g aspirin or vehicle for 30 min **(A-B)** or were allowed to swim in aspirin-supplemented water (250 mg/ml) for 24 hours **(C-D)**, followed by thrombin generation measurements. **(A,C)** Thrombin generation curves are shown. **(B,D)** Results are expressed as the percentage inhibition compared to a control group of vehicle-treated fish (n = 5). The percentage CV is indicated for all parameters. *Mann-Whitney U-test as compared to controls.

Aspirin is known to impair platelet aggregation via inhibition of platelet thromboxane A2 synthesis, thereby reducing thrombus formation. When zebrafish were treated with 30 μg/g aspirin, a trend towards a delayed and reduced thrombin generation could be observed (Fig 3A and 3B). Statistical significance could not be reached, probably due to the limited number of zebrafish. Alternatively, when fish were allowed to swim in aspirin-supplemented water (250 mg/l) for 24 hours, a delayed thrombin generation could be observed as evident from the lag time which was significantly longer than in controls (Fig 3C and 3D).

### SEM analysis of fibrin clots

**Fig 4:**
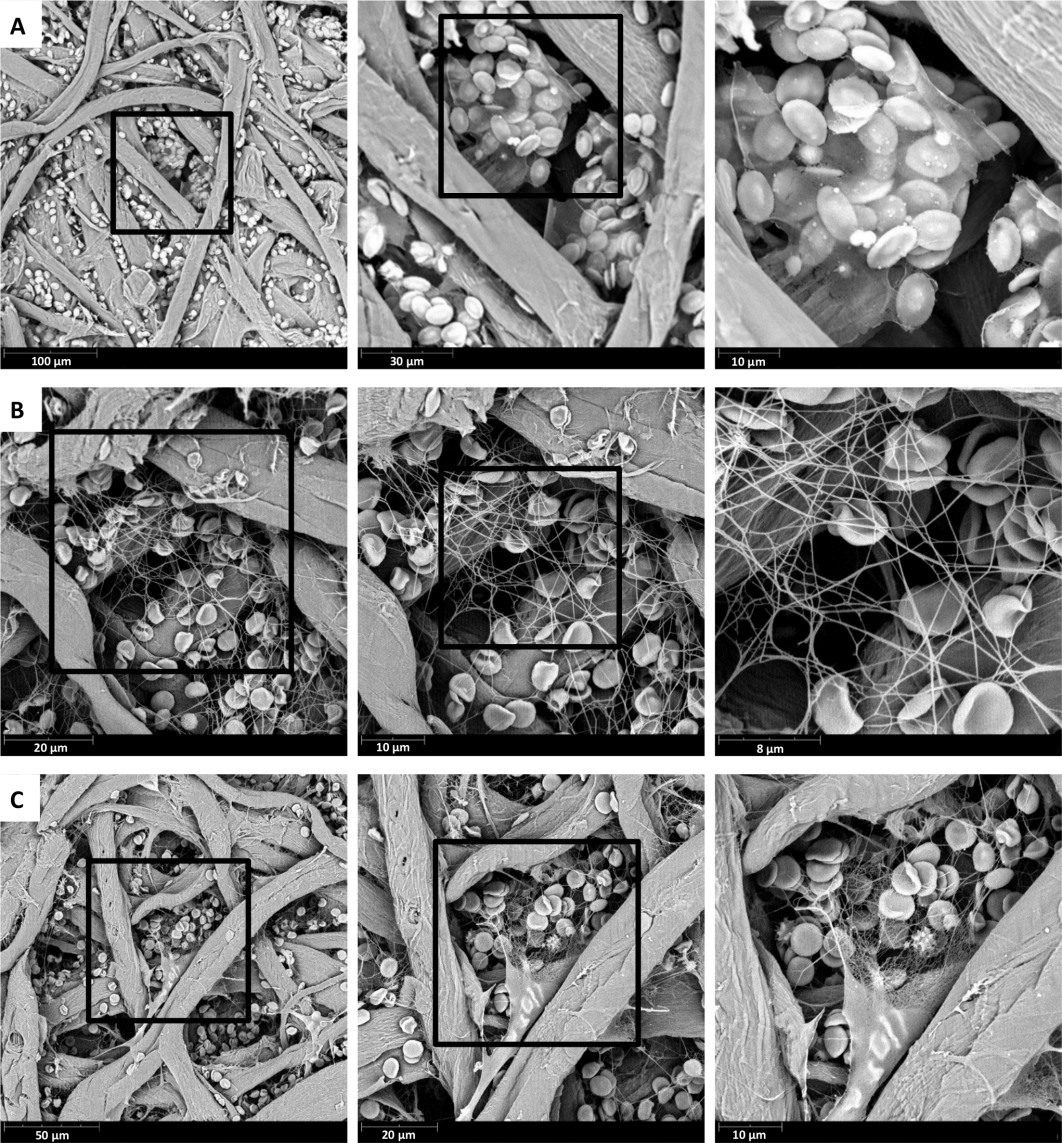
Scanning electron microscopy (SEM) of blood clots. Representative image of SEM analysis of blood clots formed during thrombin generation measurements. Sequential enlargements of the fibrin network with entrapped red blood cells are depicted. **(A)** Zebrafish blood clot. **(B)** Human blood clot without added tissue factor (TF). **(C)** Human blood clot with addition of a high dose (100 pM) of TF.

Given the accelerated and increased thrombin generation in zebrafish compared to humans, we decided to analyze the fibrin ultrastructure at the end of a thrombin generation experiment by SEM. The mineral oil that was used to prevent evaporation was removed and the fibrin that was formed was fixated for visualization with SEM. Representative images of the fibrin network are shown in Fig 4. Analysis of the zebrafish clot (Fig 4A) revealed a much denser network compared to human clots obtained from finger prick blood without the addition of tissue factor (Fig 4B), composed of thick fibrin fibers. Interestingly, the structure of the network obtained from fish blood is more comparable to a human fibrin network triggered with high tissue factor concentrations (Fig 4C). Much thinner fibrin fibers can be observed which are arranged in dense networks. Red blood cells and smaller cells we suspect could be thrombocytes, but are smaller than would be expected, are entrapped in the zebrafish fibrin network. This might suggest their participation in the process of coagulation.

## Discussion

In this study, we optimized an assay to measure thrombin generation in whole blood obtained from a single zebrafish. A major advantage compared to previous tests, is the use of whole blood instead of plasma, resulting in a quick and reliable screening tool to measure blood coagulation in zebrafish. Furthermore, as the measurement starts directly after the blood collection, the use of an anti-coagulant that may influence coagulation is avoided.

The suitability of the zebrafish model to study hemostasis depends hugely on the degree of similarity between the zebrafish and mammalian systems. Previous studies have established that, despite the evolutionary distance, the major coagulant and anti-coagulant pathways are similar between zebrafish and mammals, as evident from the presence of a comparable contact activation system, extrinsic pathway and common pathway [4]. The effects of prothrombin deficiency in zebrafish embryos, ranging from early morphological defects and internal bleeding to occasional bleeding in the brain at a later stage, suggest that the mechanism of thrombin signaling is conserved across vertebrates [15]. To our knowledge, our method is the first that allows measurement of thrombin generation in whole blood obtained from a single zebrafish. Thrombin generation experiments in zebrafish showed a similar inter-individual variation as in humans [12]. However, thrombin generation proved to be significantly accelerated and increased compared to humans, even if high amounts of tissue factor were added to the human blood. With the current blood collection method, using a pair of scissors, exposure of the blood to tissue factor is inevitable. Therefore the current method is best suited for tests related to the intrinsic coagulation system. As previously described for human plasma [14], a lower temperature (28°C, physiological temperature for zebrafish) resulted in slower and higher thrombin generation as compared to higher temperature (37°C) in zebrafish blood. Due to practical considerations and because the adapted PT and APTT for zebrafish plasma by Jagadeeswaran *et al*. are performed at 37°C [4], we decided to perform all subsequent experiments at 37°C.

By pre-treating zebrafish with thrombin inhibitors, we clearly provided evidence that the observed fluorescent signals in our assay are the result of thrombin. Pre-treatment of zebrafish with aspirin resulted in a tendency towards a delayed and reduced thrombin generation. The treatment with melagatran, hirudin and rivaroxaban was found to significantly inhibit thrombin generation in zebrafish. Hirudin microinjections into early zebrafish embryos were previously shown to inhibit fibrin forming activity and to cause abnormal development, suggesting a role for thrombin in early development [16]. Furthermore, Jagadeeswaran *et al*. demonstrated that warfarin has similar effects in zebrafish compared to mammals, illustrating that vitamin K-dependent pathways in fish are comparable to human pathways [4,8]. Interestingly, as all tested anticoagulant therapies were found to diminish thrombin generation in zebrafish, zebrafish may be used as an *in vivo* model system to test the pharmacokinetic and pharmacodynamic aspects of (novel) therapeutics on thrombin generation.

As to the fibrin formation, the appearance of a dense fibrin network composed of thin fibrin fibers is in line with the accelerated and increased thrombin generation. Indeed, high versus low concentrations of thrombin lead to dense networks of thin fibers versus permeable clots composed of thick woven fibrin strands, respectively [9,17,18]. Although addition of high amounts of tissue factor to human blood resulted in an accelerated thrombin generation and denser fibrin network, the same velocity index of thrombin generation and fibrin density as the zebrafish clots could not be reached. This suggests that other (unknown) factors play a role. Since fish live in an aqueous environment, wounds should be sealed off very fast for the animal to survive. The accelerated thrombin generation and dense fibrin network formation is therefore evolutionary favorable.

In conclusion, we developed a fast and reliable method to measure thrombin generation in whole blood collected from a single zebrafish. Given the huge similarities between coagulation pathways of zebrafish and mammals, zebrafish may be an ideal *in vivo* model to determine the effect of novel therapeutics on thrombin generation. Additionally, because of the ease with which gene functions can be silenced, zebrafish may serve as a model organism for further mechanistical research in thrombosis and hemostasis.
